# Reduction of Gold (III) and Tellurium (IV) by *Enterobacter cloacae* MF01 Results in Nanostructure Formation Both in Aerobic and Anaerobic Conditions

**DOI:** 10.3389/fmicb.2018.03118

**Published:** 2018-12-18

**Authors:** Fernanda Contreras, Esteban Vargas, Karla Jiménez, Claudia Muñoz-Villagrán, Maximiliano Figueroa, Claudio Vásquez, Felipe Arenas

**Affiliations:** ^1^Laboratorio Microbiología Molecular, Departamento de Biología, Facultad de Química y Biología, Universidad de Santiago de Chile, Santiago, Chile; ^2^Center for the Development of Nanoscience and Nanotechnology, Santiago, Chile; ^3^Departamento de Ciencias Básicas, Facultad de Ciencias, Universidad Santo Tomás, Santiago, Chile

**Keywords:** metal(loid)s, *Enterobacter cloacae*, metal(loid) resistance, EPS, reduction, aerobiosis, anaerobiosis, nanostructures

## Abstract

Microorganism survival in the presence of toxic substances such as metal(loid)s lies chiefly on their ability to resist (or tolerate) such elements through specific resistance mechanisms. Among them, toxicant reduction has attracted the attention of researchers because metal(loid)-reducing bacteria are being used to recover and/or decontaminate polluted sites. Particularly, our interest is to analyze the toxicity of gold and tellurium compounds for the environmental microorganism *Enterobacter cloacae* MF01 and also to explore the generation of nanostructures to be used in future biotechnological processes. Resistance of *E. cloacae* MF01 to gold and tellurium salts as well as the putative mechanisms involved -both in aerobic and anaerobic growth conditions- was evaluated. These metal(loid)s were selected because of their potential application in biotechnology. Resistance to auric tetrachloride acid (HAuCl_4_) and potassium tellurite (K_2_TeO_3_) was assessed by determining areas of growth inhibition, minimum inhibitory concentrations, and growth curves as well as by viability tests. *E. cloacae* MF01 exhibited higher resistance to HAuCl_4_ and K_2_TeO_3_ under aerobic and anaerobic conditions, respectively. In general, their toxicity is mediated by the generation of reactive oxygen species and by a decrease of intracellular reduced thiols (RSH). To assess if resistance implies toxicant reduction, intra- and extra-cellular toxicant-reducing activities were evaluated. While *E. cloacae* MF01 exhibited intra- and extra-cellular HAuCl_4_-reducing activity, tellurite reduction was observed only intracellularly. Then, Au- and Te-containing nanostructures (AuNS and TeNS, respectively) were synthesized using crude extracts from *E. cloacae* MF01 and their size, morphology, and chemical composition was evaluated.

## Introduction

Although most of the dry weight of a living cell is composed by elements such as C, O, H, N, P and S, there are other elements that are required to sustain independent life (Nies and Silver, 1995). Among them, some metal(loid)s fulfill cellular functions that cannot be carried out by organic molecules, thus being crucial for the structure of cell membranes, DNA and proteins; they also participate in a number of key cellular processes such as electron transfer and catalysis (Lemire et al., 2013). Nevertheless, there are other metal(loid)s for which there is no known biological function to date (Nies, 1999; Harrison et al., 2004). These include gold (Au^3+^), mercury (Hg^2+^), cadmium (Cd^2+^) and tellurium (Te^4+^, Te^6+^), which are extremely toxic to organisms even at very low concentrations (Nies, 1999; Taylor, 1999; Pérez et al., 2007; Zannoni et al., 2007; Lemire et al., 2013). Despite their toxicity, the interest in these elements has grown the last few years because of its applicability in the medical, metallurgical, chemical, and optical industry (Bao et al., 2010).

In general terms, under aerobic conditions metal(loid) toxicity is due -at least in part- to the increased generation of intracellular reactive oxygen species (ROS), which can affect a number of key metabolic pathways generating: (i) protein and enzyme dysfunction, oxidation of catalytic residues and [Fe-S] cluster dismantling (Stadtman and Levine, 2000; Helbig et al., 2008); (ii) single or double breaks in DNA (Imlay et al., 1988; Touati et al., 1995); (iii) lipid peroxidation of cell membranes (Hong et al., 2012; Lemire et al., 2013), among others. Since ROS are formed only in aerobic conditions (Imlay, 2003), the observed toxicant damage in anaerobiosis should be independent of ROS.

Microbes have evolved a number of strategies to thrive in the presence of these toxic metal(loid)s, which are rather specific: no common resistance strategy has been described to date (Lemire et al., 2013). The referred strategies include: (i) decreased uptake or enhanced efflux of metal ions because of changes in the activity or production of membrane transport proteins (Ma et al., 2009); (ii) intra- or extra-cellular sequestration, mediated mainly by polymers or siderophores that can trap or precipitate metal ions (Harrison et al., 2007; Zannoni et al., 2007; Dimkpa et al., 2008); (iii) chaperone-, enzyme- or antioxidants-mediated repair of molecules that are prone to oxidation by metals; (iv) metabolic bypass, by which an alternative metabolic pathway is generated to cope with the metal ion (Lemire et al., 2010) and finally, (v) chemical modification of the toxicant, that usually converts the metal(loid) into a less toxic (or less available) form (Silver and Phung, 1996, 2005).

The phenomenon of metal(loid) reduction has also been observed *in vitro* using crude cell extracts or purified proteins. In this frame, a number of enzymes involved in metal(loid) tolerance have been identified which, apart from their normal metabolic functions can help in the process of metal reduction. In particular, some of them belong to the flavoprotein family (Heuts et al., 2009) including the E3 component of the multienzyme pyruvate dehydrogenase complex of *Escherichia coli* and other microorganisms (Castro et al., 2008), NDH-II of *E. coli* (Díaz-Vásquez et al., 2015), thioredoxin reductase (Trx) and alkyl hydroperoxide reductase (AhpF) of *Staphylococcus haemolyticus* BNF01 (Arenas-Salinas et al., 2016) and glutathione reductase (GorA) from *E. coli*; all of them can generate reduced, elemental tellurium while GorA also produces metallic gold from tetrachloroauric acid reduction (Pugin et al., 2014; Figueroa et al., 2018).

On the other hand, other groups have suggested that extracellular polymeric substances (EPSs) could also participate in metal(loid) reduction (Smith and Gadd, 2000; Dohnalkova et al., 2005; Harish et al., 2012). EPS can be defined as a matrix that forms part of the bacterial cell surface which is composed by a variety of macromolecules including polysaccharides, proteins, nucleic acids, phospholipids, and other low molecular weight components (Wingender et al., 1999). The mechanism(s) of EPS-mediated metal(loid) reduction has been hard to determine because EPS constituents vary greatly among microorganisms (Pal and Paul, 2008).

Microorganisms can remediate metal(oid)s because of their ability to change the compound’s oxidation state, influencing their solubility that results in the formation of less toxic and often insoluble derivatives (Suresh, 2012). In some cases, these represent metallic, nanoscaled arrangements generically known as nanostructures (NS). Reports on tellurium (Arenas-Salinas et al., 2016) and gold (Correa-Llantén et al., 2013) nanostructures synthesized by *E. coli* and *Geobacillus* sp., respectively, have generated great interest due to its potential application in the field of biotechnology. Nanostructure production by biological methods is considered a safe, economical and environmentally friendly process, in contrast to chemical procedures that require high temperatures, anaerobic conditions and/or the presence of a number of toxic components (Turner et al., 2012).

The specific, mechanical, optical and fluorescence properties exhibited by metal(loid)-containing nanostructures makes them excellent candidates to be used in the field of physics, chemistry, electronics, materials science, biomedicine, electronics and agriculture, among others (Thakkar et al., 2010). Particularly interesting is the field of biomedicine, where NS have been used in therapies to detect cancer (Rao, 2008) and as antibacterials (Amstad et al., 2011; Azam et al., 2012). In this line, nanostructures with bactericidal (Cui et al., 2012; Xiu et al., 2012; Pugin et al., 2014; Oves et al., 2017) as well as antibacterial activity have been reported (Cioffi and Rai, 2012).

Summarizing, it is of great interest to unveil the mechanism by which microorganisms are able to tolerate metal(loid) since it could involve the reduction of these elements to less toxic forms. In this line, this work describes the generation of Au- and Te-containing NS by *Enterobacter cloacae* MF01 which could represent interesting candidates for biotechnological processes.

## Materials and Methods

### Bacterial Isolation and Growth Conditions

*Enterobacter cloacae* MF01 was isolated from environmental water samples from central Chile (Cascada Invertida, Maule Region; Figueroa et al., 2018). The bacterium was grown in LB medium with shaking at 150 rpm at 37°C both in aerobic or anaerobic conditions (100% N_2_, Coy chamber, Coy Laboratory Products, Inc.). Growth in solid media was carried out in 2% (w/v) LB agar plates.

### Determination of the Growth Inhibition Area

One hundred μL of cells grown to stationary growth phase were evenly spread on LB agar plates. After drying briefly in air, three sterile filter paper disks (0.6 cm diameter) were placed on each plate, to which 10 μL of the toxican to be tested were added: 50 mM auric tetrachloride acid (HAuCl_4_) or 4 mM potassium tellurite (K_2_TeO_3_). The zone of growth inhibition (cm^2^) was determined after incubating the plates at 37°C for 24 h.

### Determination of the Minimal Inhibitory Concentration (MIC)

Serial dilutions (1:2) were made from sterile solutions of HAuCl_4_ and K_2_TeO_3_ in 1 mL of LB medium in 48-well culture plates. Subsequently, 10 μL of *E. cloacae* MF01 cultures grown in LB up to OD_600_ 0.6 (aerobic conditions) or 0.4 (anaerobic conditions) were added to each well and incubation proceeded with constant shaking at 37°C. Minimal inhibitory concentrations (MICs) were determined by monitoring turbidity at 600 nm after 24 h.

### Curves and Growth Parameters

Overnight cultures were diluted 1:100 with fresh LB medium and incubated at 37°C to OD_600_ ∼0.6 (aerobiosis) or 0.4 (anaerobiosis). Then, 10 μL were added to 1 mL of fresh LB medium containing sublethal concentrations of each metal(loid) in 48-well plates. Growth was monitored at 600 nm every 30 min at 37°C for 15 h in a TECAN Infinite^®^ M200 plate reader.

Data were plotted and growth parameters corresponding to the maximum OD_600_ (%), lag phase duration (h), growth rate (μ) and generation time (h) were determined. The maximum OD_600_ reached by the culture was referred to that of the control (100%). The duration of the lag phase was determined as the end point of the latency phase and beginning of the exponential phase of bacterial growth. The growth rate was estimated as the change in the OD_600_ per h during the exponential growth phase. Finally, the generation time was calculated from the equation g = 0.693/μ.

### Bacterial Viability Assays

Overnight cultures of *E. cloacae* MF01 were diluted 1:100 with fresh LB medium and incubated at 37°C and 150 rpm to OD_600_ 0.6 (aerobic) or 0.4 (anaerobic). Subsequently, 180 μL aliquots of the cultures were added to 20 μL of fresh LB medium containing different concentrations of the toxicants in 96 well plates (0–38 mM HAuCl_4_, 0–60 mM K_2_TeO_3_). After 15 min, aliquots of 20 μL were taken and added to a 96-well plate containing 180 μL of 0.9% NaCl. Serial dilutions (1:10) were made in the 96-well plate, from which 4 μL were seeded in LB agar plates (drop). The number of colony forming units (CFUs) was determined after incubating at 37°C for 24 h.

### Detection of Total Reactive Oxygen Species (Total ROS)

Intracellular total ROS levels in cells exposed to HAuCl_4_ or K_2_TeO_3_ were determined using 2′,7′-dichlorodihydrofluorescein diacetate as probe (H_2_DCFDA, Calbiochem). Overnight *E. cloacae* MF01 cultures were diluted 1:100 with fresh LB medium and incubated at 37°C with shaking to OD_600_ ∼0.6 (aerobic) or ∼0.4 (anaerobic). Then, cells were treated with the toxicants (0.5x of the corresponding MIC) and with ascorbic acid (10 mM). One mL aliquots were taken at different times (0, 15, and 120 min) and sedimented at 12,000 ×*g* for 5 min. Pellets were washed three times with 50 mM Tris-HCl buffer pH 7.4 and subsequently, 50 μL were added to 930 μL of 50 mM Tris-HCl buffer pH 7.4, treated with H_2_DCFDA (40 μM, prepared in dimethyl sulfoxide) and incubated in the dark for 30 min. Cells were centrifuged as above for 5 min and washed with 50 mM Tris-HCl buffer pH 7.4. Finally, 200 μL of the cell suspension were used to determine fluorescence intensity in a Tecan Infinite^®^ M200 Pro plate multi lector (excitation 490 nm, emission 527 nm). Fluorescence intensity was normalized by OD_600_. Per cent of fluorescence intensity regarding that of the control condition (100%) was calculated.

### Determination of the Reduced Cellular Thiol (RSH) Concentration

Overnight cultures of *E. cloacae* MF01 were diluted 1:100 with fresh LB medium and incubated at 37°C to ∼OD_600_ 0.6 (aerobic) or 0.4 (anaerobic). Cultures were treated with the toxicants as above and then aliquots of 500 μL were taken at different times (0, 15 min and 24 h) and sedimented at 12,000 ×*g* for 2 min. Sediments were suspended in 1 mL of 5 mM EDTA, 0.1% SDS, 0.1 mM DTNB and 50 mM Tris-HCl buffer pH 8.0. The suspension was incubated for 30 min at 37°C and subsequently, vigorously shaken and sedimented at 12,000 ×*g* for 10 min. Finally, the supernatant was rescued and the absorbance at 412 nm measured in a Tecan Infinite^®^ M200 Pro plate multireader. The RSH content was calculated through a calibration curve constructed with GSH standards (0–200 μM). RSH concentration was normalized by protein concentration. Per cent of RSH was calculated with respect to the respective control condition (100%).

### Protein Concentration

Protein concentration was determined by the method described by Bradford (1976) using bovine serum albumin (BSA) as standard.

### Determination of Gold and Tellurium Total Content by Optical Emission Spectroscopy – Inductively Coupled Plasma (ICP-OES)

*Enterobacter cloacae* was grown to OD_600_ ∼0.6 (aerobic) or 0.4 (anaerobic), treated with the toxicants as above for 15 min and centrifuged at 12,000 ×*g* for 4 min at 4°C. After storing the supernatant, the sediment was suspended in 50 mM Tris-HCl pH 7.4 buffer supplemented with 0.1 mM phenylmethylsulfonyl fluoride (PMSF, Life Technologies, Inc.). After sonicating on ice (four pulses of 20 s each), the cell debris was removed by centrifugation (21,000 ×*g*) for 10 min at 4°C. The supernatant was ultracentrifuged at 120,000 ×*g* for 1 h in a Beckman optima LE-80K centrifuge using a 70.1 Ti rotor. This new supernatant was considered the crude extract while the sediment represented the membrane fraction.

Supernatants, intracellular and membrane fractions from cells exposed to the toxicants were diluted with HNO_3_ (up to 2%), filtered (0.22 μm nylon filters, GE Healthcare, Life Sciences) and used for determining gold and tellurium in a PerkinElmer Optima 2000 DV ICP-OES device. Calibration curves were made with gold and tellurium commercial standards. Per cent of gold and tellurium was calculated regarding the respective controls (100%, aerobic and anaerobic).

### Metal(loid)-Reducing Activity by Crude Extracts

HAuCl_4_ and K_2_TeO_3_ reducing activities were assessed at 37°C using 20 mM potassium phosphate (pH 6.0–7.0) and 50 mM Tris-HCl (pH 8.0–9.0) buffers, respectively. Generation of metallic gold and tellurium was monitored in a TECAN Infinite M200 Pro multimode plate reader at 500 nm (Te, Pugin et al., 2014) and 540 nm (Au, Correa-Llantén et al., 2013). Reactions were carried out in a final volume of 200 μL that contained the appropriate buffer, 1 mM HAuCl_4_ or K_2_TeO_3_, 1 mM NADH or NADPH and the crude extract. Controls excluding extracts were run in each case to rule out abiotic reduction. For tellurite reduction, the reaction mix also contained 1 mM β-mercaptoethanol. An enzyme unit (U) was defined as the amount of enzyme that increased the absorbance by 0.001 units in 1 min under the assay conditions. The enzymatic activity was normalized by the protein concentration.

### Purification of Extracellular Polymeric Substances (EPSs)

Purification of bacterial EPS was accomplished by using the modified cold ethanol precipitation method described by Bramhachari and Dubey (2006). Cells of *E. cloacae* MF01 were grown to stationary phase and centrifuged at 4°C. Supernatants were filtered under vacuum using a 0.45 μm nitrocellulose filter (Merck Millipore). EPS were precipitated by adding three volumes of cold ethanol to the filtrate. After incubating at 4°C, the sample was sedimented at 12,000 ×*g* for 10 min at 4°C and washed three times with 70% ethanol. EPS were dissolved in 2 mL of miliQ water and dialyzed for 24 h at 4°C against nanopure water (dialysis membrane 12–14 kDa MW cut-off, Spectra/Por^®^).

### EPS Analysis

Total sugar content in EPS was estimated using the modified UV-sulfuric acid method of Albalasmeh et al. (2013). Briefly, 300 μL of EPS solution were mixed with 1 mL of concentrated H_2_SO_4_. The mixture was cooled on ice for 2 min and the production of furfural derivatives was monitored at 315 nm. A D-glucose calibration curve was used as standard. Protein content was determined as described in Section “Materials and Methods.”

### *E. cloacae* EPS-Mediated Metal(loid) Reducing Activity

Extracellular polymeric substance-mediated metal(loid) reducing activity was assessed as specified in Section “Metal(loid)-Reducing Activity by Crude Extracts.” The reaction was carried out in a final volume of 200 μL that contained the appropriate buffer, 1 mM HAuCl_4_ or K_2_TeO_3_, 5 mg/mL BPN’ subtilisin (Sigma) or 1 U/μL benzonase nuclease (Novagen) and 10 μL of the purified EPS. Controls to rule out abiotic reduction were run in each case. Reducing activities of HAuCl_4_ or K_2_TeO_3_ were defined as the difference in absorbance after incubation at 37°C for 24 h at 540 or 500 nm, respectively.

### Analysis of EPS by Scanning Electron Microcopy

To confirm the formation of exopolysaccharide material after toxicant exposure, cultures of *E. cloacae* MF01 were treated with 8 μM K_2_TeO_3_ (0.5x the MIC in aerobic conditions) and incubated at 37°C for 24 h. After plating on LB agar plates, incubation was continued under the same conditions for 24 h. Samples were fixed by mild heating in glass covers and then dried for later visualization. Prior to imaging, samples were stained, mounted on conductive adhesive and sputter coated with gold film. Electron microscopy analysis was performed using a SEM Zeiss EVO MA-10 equipment and imaged at an accelerating voltage of 20 kV and 8 mm of working distance. This study was conducted at the Center for the Development of Nanoscience and Nanotechnology – CEDENNA, Universidad de Santiago de Chile (USACH).

### *In vitro* Synthesis of Nanostructures

Crude extracts from *E. cloacae* MF01 (200 μg/mL protein) were used to produce nanostructures by incubation with 1 mM HAuCl_4_ or K_2_TeO_3_ for 24 h at the optimal conditions of temperature, pH, cofactor, and growth conditions (as determined from reduction assays) in a final volume of 1 mL. Controls to rule out abiotic synthesis were run in each case. Concentrations of these nanostructures were calculated by dry weight, which was expressed as μg/ml.

### Analysis of Nanostructures by Transmision Electron Microscopy

The morphology of the synthesized nanostructures was analyzed by TEM using a Hitachi Transmission Electron Microscope HT7700 (CEDENNA, USACH). To do this, a drop of the synthesized nanostructures was placed on a copper grid and analyzed briefly after.

### Energy Dispersive X-ray Spectroscopy (EDS)

Energy dispersive X-ray spectroscopy (EDS) analysis was carried out to determine the chemical composition of the synthesized nanostructures. Samples were analyzed by scanning electron microscopy (SEM) using a Zeiss EVO MA-10 microscope with a tungsten filament gun and by EDX spectra which were collected using an Oxford Instruments X-act system attached to a microscope equipped with a Penta FET Precision detector (CEDENNA, USACH). Prior to imaging, samples were supported on glass slides and stained with Gram-Hucker kit, fixed on conductive adhesive over pin stub mount and sputter coated with gold film to protect the surface from damage and calcinations and to minimize charge related artifacts. The samples were imaged at an accelerating voltage of 20 kV and 8 mm of working distance.

### Data Analysis

Plots and statistical analyses were carried out using the GraphPad Prism 7.0 (GraphPad Software, Inc.). Analysis of variance (ANOVA) and *t-*test were used considering *p* < 0.05. The statistical significance was indicated as follows: ^∗^*p* < 0.05, ^∗∗^*p* < 0.01, ^∗∗∗^*p* < 0.001, and ^∗∗∗∗^*p* < 0.0001; ns, not significant.

## Results

### Metal(loid) Resistance in *E. cloacae* MF01

Minimal inhibitory concentrations of HAuCl_4_ and K_2_TeO_3_ for *E. cloacae* MF01 both under aerobic and anaerobic conditions were determined. Under aerobic conditions, the MIC of HAuCl_4_ was twice that in the absence of oxygen (0.50 vs. 0.25 mM), thus suggesting that the anaerobic condition makes the isolate more sensitive to the toxicant. Conversely, TeO_3_^2-^ was more toxic for *E. cloacae* MF01 in aerobic growth conditions (0.016 vs. 0. 031 mM).

Supplementary Figure S1 shows the areas of growth inhibition of *E. cloacae* MF01 exposed to HAuCl_4_ or K_2_TeO_3_ under aerobic and anaerobic growth conditions. While the bacterium grew better in the presence of HAuCl_4_ in aerobiosis, it turned to be more resistant to tellurite in the anaerobic condition.

**FIGURE 1:**
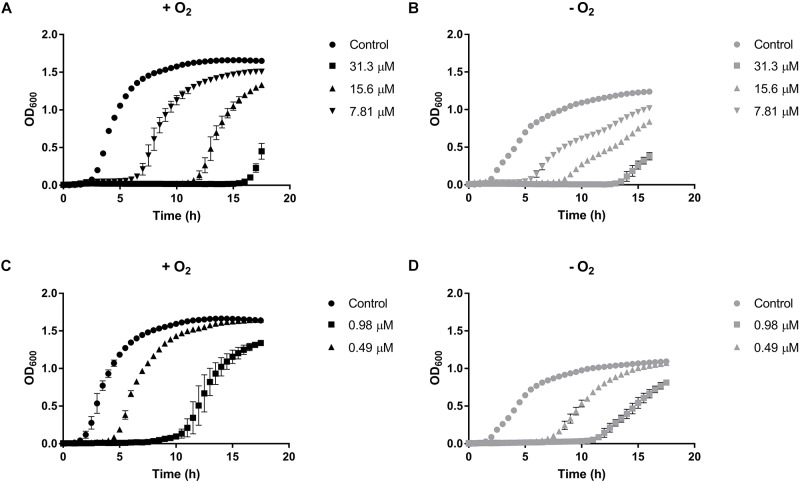
Growth curves of *Enterobacter cloacae* MF01 exposed to sublethal concentrations of HAuCl_4_ or K_2_TeO_3_ under aerobic and anaerobic conditions. Cells were grown in LB medium in the absence or presence of HAuCl_4_ **(A,B)** or K_2_TeO_3_ **(C,D)** both under aerobic **(A–C)** and anaerobic conditions **(B,D)**. Each point corresponds to the average of six independent tests ± SD.

The effect of sublethal concentrations of HAuCl_4_ and K_2_TeO_3_ on *E. cloacae* MF01 growth was evaluated in aerobiosis and anaerobiosis (Figure 1). The bacterium showed less and slower growth -irrespective of the presence of oxygen- than the respective controls in the presence of HAuCl_4_ (15.6 and 7.8 μM, Figures 1A,B) or K_2_TeO_3_ (0.98 and 0.49 μM, Figures 1C,D).

The variation of *E. cloacae* growth parameters when exposed to HAuCl_4_ or K_2_TeO_3_ was quantified (Supplementary Table S1). When cells were exposed to defined HAuCl_4_ or K_2_TeO_3_ concentrations, the maximal OD_600_ was always higher in aerobic conditions (Figure 1 and Supplementary Table S1). Lag phase duration in the presence of HAuCl_4_ was lower in anaerobic conditions while the opposite situation was observed upon tellurite exposure (Supplementary Table S1). In turn, in the presence of either toxicant the growth rate showed a trend to be higher in the aerobic condition. A similar result was observed when generation time was calculated, i.e., *E. cloacae* MF01 exposed to HAuCl_4_ or K_2_TeO_3_ exhibited a shorter generation time in aerobiosis (Supplementary Table S1).

Viability tests of *E. cloacae* MF01 exposed to defined concentrations of HAuCl_4_ or K_2_TeO_3_ for 15 min in aerobiosis or anaerobiosis showed that the bacterium tolerates higher toxicant concentrations in the presence of oxygen. The aerobic LC_50_ of HAuCl_4_ and K_2_TeO_3_ was 5.9 ± 1.084 and 5.1 ± 1.09 mM, respectively, while under anaerobic conditions the LC_50_ of HAuCl_4_ and K_2_TeO_3_ was 4.8 ± 1.048 and 0.50 ± 0.102 mM, respectively.

### Toxicity of HAuCl_4_ and K_2_TeO_3_ for *E. cloacae* MF01

**FIGURE 2:**
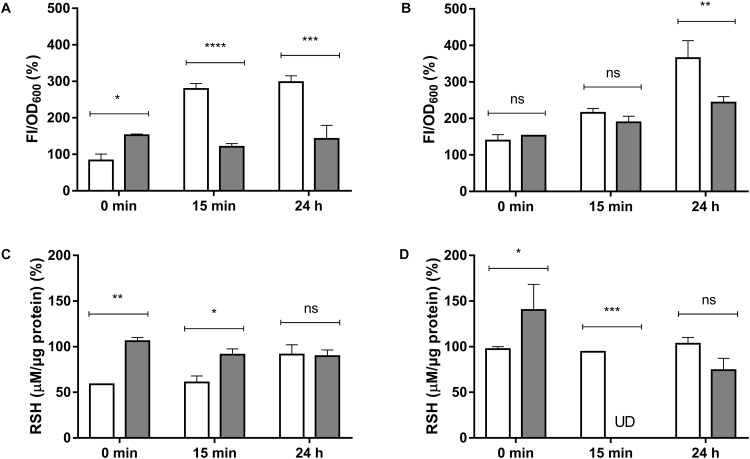
Total ROS **(A,B)** and intracellular RSH **(C,D)** concentration in *E. cloacae* MF01 exposed to HAuCl_4_ or K_2_TeO_3_. Cells were treated for the indicated times with HAuCl_4_ **(A–C)** or K_2_TeO_3_ **(B,D)**, in aerobic (white bars) or anaerobic conditions (gray bars). While HAuCl_4_ exposure concentrations were 0.25 mM (aerobic) and 0.125 mM (anaerobic), those of K_2_TeO_3_ were 0.008 and 0.016 mM, respectively. Fluorescence intensity and RSH concentrations were expressed as per cent of those values observed in the respective control conditions (100%). The data represent the average of three independent tests ± SD. ^∗^*p* < 0.0216; ^∗∗^*p* < 0.0012; ^∗∗∗^*p* < 0.0002; ^∗∗∗∗^*p* < 0.0001; ns, not significant; UD, undetectable.

Total ROS determination, as described in Methods, was assessed to determine if exposure of *E. cloacae* to HAuCl_4_ or K_2_TeO_3_ results in increased intracellular ROS (Figures 2A,B). While at short times exposure to HAuCl_4_ exhibited higher ROS content in anaerobiosis, after 15 min and 24 h the highest ROS content was observed in aerobic conditions (Figure 2A). Regarding tellurite exposure, significant differences in ROS content were observed only after 24 h (Figure 2B). In addition and irrespective of the presence of oxygen, ascorbic acid treatment decreased ROS production both in HAuCl_4_- or K_2_TeO_3_-exposed cultures (Supplementary Figure S2).

It was also determined if exposure to the toxicants affects intracellular RSH concentration (Supplementary Table S2, Figures 2C,D). Upon HAuCl_4_ exposure, a soft trend to RSH decrease was observed in anaerobiosis (Figure 2C). In the case of K_2_TeO_3_, RSH were almost undetectable in the absence of oxygen after 15 min of exposure (Figure 2D).

**FIGURE 3:**
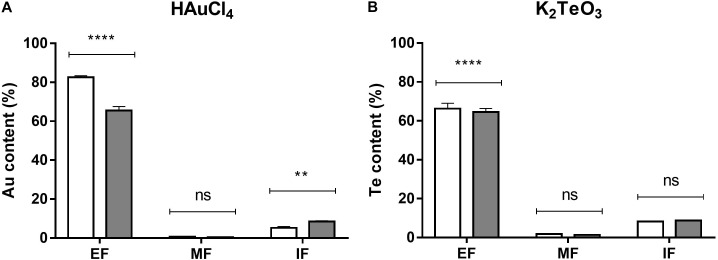
Au and Te content in *E. cloacae* MF01 exposed to the indicated toxicants under aerobic and anaerobic growth conditions. Au and Te content was determined in the extracellular (EF), membrane (MF) and intracellular fractions (IF) of bacteria exposed to HAuCl_4_ **(A)** or K_2_TeO_3_ **(B)** for 15 min in aerobic (white bars) or anaerobic (gray bars) conditions. Toxic concentrations were as indicated in the legend to Figure 2. Au and Te content are expressed as % of the initial treatment concentration. Bars represent the average of three independent tests ± SD. ^∗∗^*p* < 0.0029; ^∗∗∗∗^*p* < 0.0001; ns, not significant.

Finally, and to assess how much of the toxicant was entering the cell, intracellular HAuCl_4_ and K_2_TeO_3_ were determined by ICP-OES as total Au and Te, respectively. Figure 3 shows that almost all the metal(loid) remains in the extracellular fraction.

### Gold and Tellurite Reduction by *E. cloacae* MF01 Cell Extracts

**FIGURE 4:**
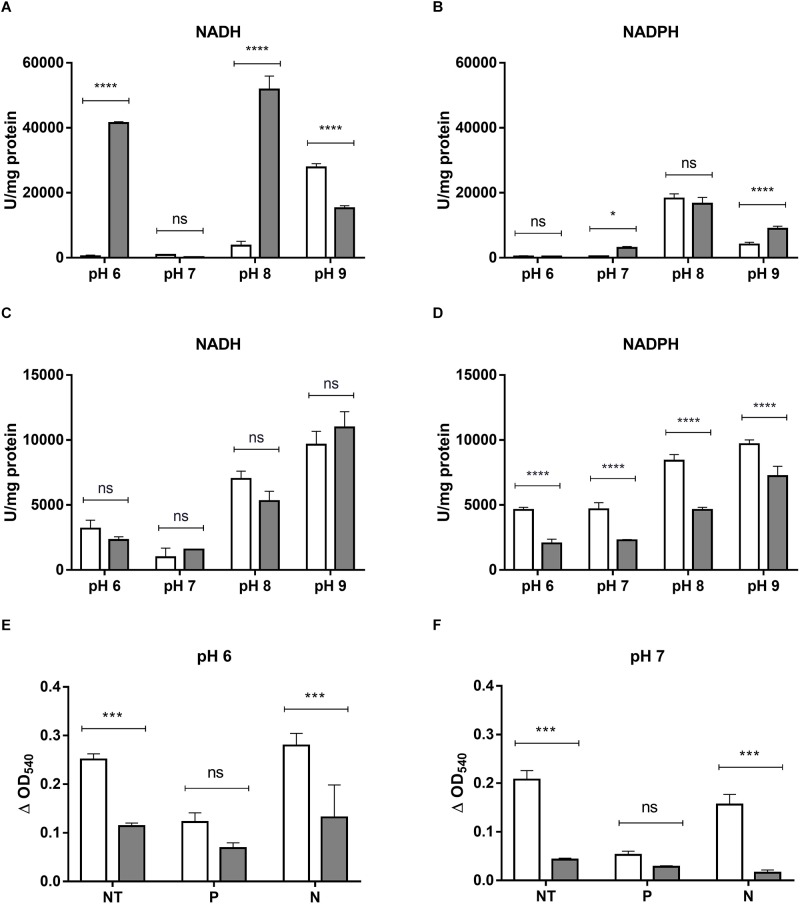
HAuCl_4_ and K_2_TeO_3_ reducing activity by crude extracts or EPS from *E. cloacae* MF01. HAuCl_4_ **(A,B)**- or K_2_TeO_3_ **(C,D)**-reducing activity by crude extracts of *E. cloacae* MF01 was assessed either with NADH or NADPH, at pH values 6.0, 7.0, 8.0 and 9.0, under aerobic (white bars) and anaerobic conditions (gray bars). Assays for HAuCl_4_-reducing activity mediated by EPS **(E,F)** were carried out using samples without treatment (NT) and with previous protease (P) or nuclease (N) treatments, at the indicated pH values and under aerobic (white bars) or anaerobic conditions (gray bars). Bars represent the average of three independent tests ± SD. ^∗^*p* < 0.05; ^∗∗∗^*p* < 0.0002; ^∗∗∗∗^*p* < 0.0001; ns, no significant.

The ability of crude extracts from *E. cloacae* MF01 to reduce HAuCl_4_ and K_2_TeO_3_ was analyzed under aerobic and anaerobic growth conditions, different pH values and electron donors (Figure 4). The extract showed the highest gold-reducing activity at pH 8.0 using NADH as cofactor (Figure 4A). However, this activity was measurable at most pH values, irrespective of the electron donor used (Figures 4A,B); the exception was pH 7.0/NADH and pH 6.0/NADPH. On the other hand, the extract showed tellurite reductase (TR) activity at all pH values, cofactors and the presence or absence of oxygen (Figures 4C,D). With NADH as cofactor, TR showed no variations among aerobic and anaerobic conditions at all pH values tested (Figure 4C). The highest TR activity was observed at pH 9.0 using NADH as electron donor. In general, TR was higher in aerobic conditions at any pH tested, using NADPH as cofactor (Figure 4D).

As reveled by SEM, *E. cloacae* MF01 produced EPSs when grown in the presence of 0.008 mM K_2_TeO_3_ (Supplementary Figures S3C,D) but not in the untreated control condition (Supplementary Figures S3A,B). This observation prompted us to analyze EPS-mediated HAuCl_4_- and K_2_TeO_3_-reducing activities in aerobic and anaerobic growth conditions (Figure 4). EPS exhibited only gold-reducing activity, which was always higher in aerobic conditions both at pH 6.0 and 7.0. Interestingly, the highest activity was observed at pH 6.0 with untreated EPS or after nuclease digestion (benzonase, Figure 4E). Protease (subtilisin BPN’) treatment decreased gold-reducing activity at pH 6.0 and 7.0 (Figures 4E,F).

The chemical composition of purified EPS from *E. cloacae* MF01 was examined and the results showed that EPS contained a higher protein concentration when purified from anaerobically-grown cells (0.28 ± 0.13 vs. 0.24 ± 0.13).

### Synthesis and Characterization of NS Synthesized Using *E. cloacae* MF01 Cell Extracts

In general, *in vitro* nanostructure synthesis by crude extracts of this bacterium lasted about 16 h. NS were generated at different pH values, electron donors and in the presence or absence of oxygen. Gold-containing nanostructures (AuNS) were synthesized in the following conditions: (i) O_2_ + NADH + pH 9.0, (ii) O_2_ + NADPH + pH 8.0, and (iii) O_2_ + NADPH + pH 9.0, (iv) -O_2_ + NADH + pH 8.0, (v) -O_2_ + NADH + pH 9.0, and (vi) -O_2_ + NADPH + pH 9.0. In turn, tellurium-containing nanostructures (TeNS) were synthesized aerobically and anaerobically in the following conditions: (i) NADH + pH 8.0, (ii) NADH + pH 9.0, (iii) NADPH + pH 8.0, and (iv) NADPH + pH 9.0. Supplementary Table S3 shows NS concentrations. The highest production of AuNS was accomplished under anaerobic conditions; those synthesized at pH 8.0/NADH exhibited a concentration of 1,600 μg/mL, while the highest production of these NS was at pH 9/NADH with a concentration of 900 μg/mL. Best conditions for TeNS production were aerobiosis, pH 9.0/NADPH (700 μg/mL).

**FIGURE 5:**
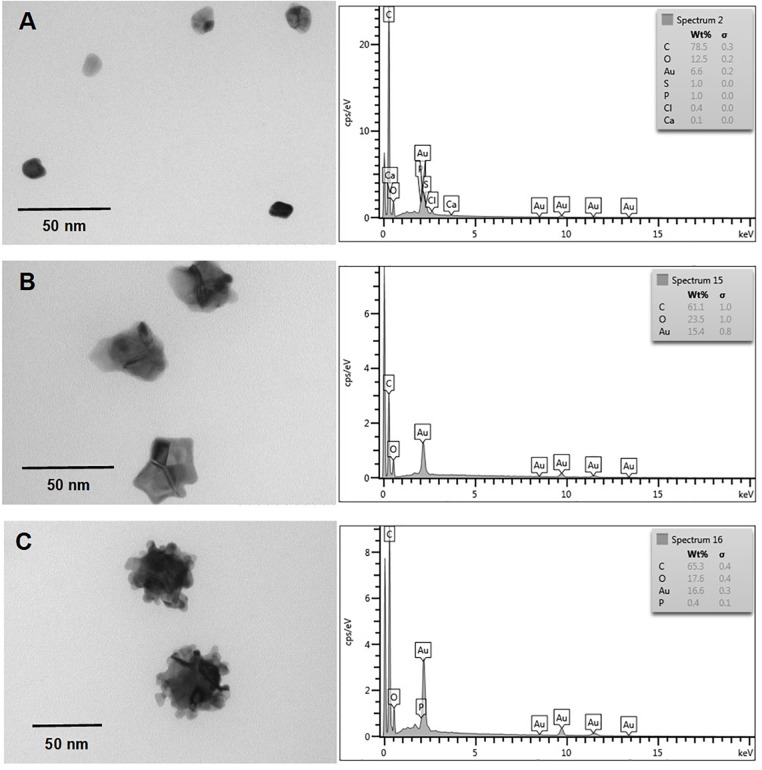
*In vitro*, aerobic synthesis of AuNS by *E. cloacae* MF01 crude extracts. Electron micrographs (left) and EDS analysis (right) show AuNS synthesized under the following experimental conditions: **(A)** NADH/pH 9.0; **(B)** NADPH/pH 8.0; **(C)** NADPH/pH 9.0.

**FIGURE 6:**
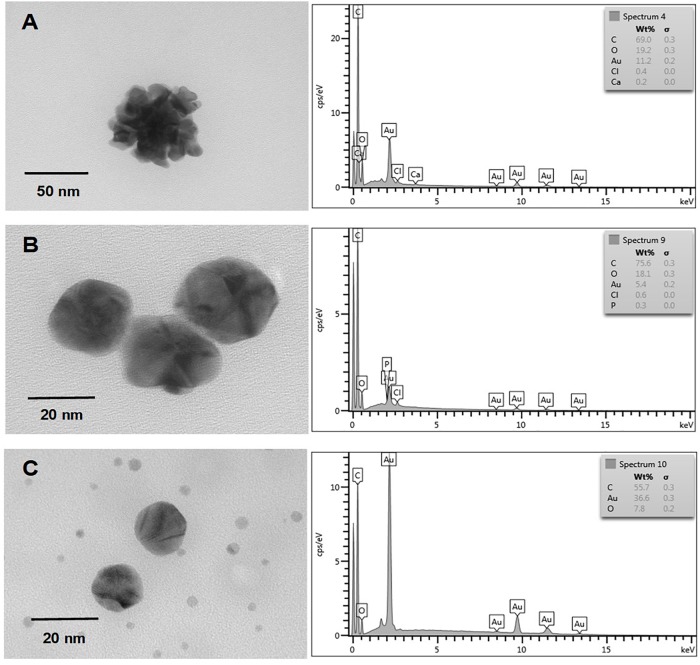
*In vitro*, anaerobic synthesis of AuNS by *E. cloacae* MF01 crude extracts. Electron micrographs (left) and EDS analysis (right) show AuNS synthesized under the following experimental conditions: **(A)** NADH/pH 8.0; **(B)** NADH/pH 9.0; **(C)** NADPH/pH 9.0.

Nanostructure and chemical composition of AuNS and TeNS were determined by transmission electron microscopy (TEM) and energy-dispersive X-ray spectroscopy (EDS), respectively. Figures 5, 6 shows AuNS synthesized under aerobic and anaerobic conditions, respectively. NS exhibited a heterogeneous morphology, including some degree of aggregation, thus forming structures of larger size. TEM showed some more electron-dense material, chiefly in the aggregated material. With the exception of those structures shown in Figure 5 (bottom panel) and 6 (upper panel), in general AuNS size was < 50 nm. Aerobically synthesized NS showed a gold content of 6.6, 15.4, and 16.5% (Figures 5A–C, respectively), and contained high amounts of carbon and oxygen. Excepting the NS shown in Figure 6C, anaerobically synthesized nanostructures contained less gold than their aerobic counterparts.

**FIGURE 7:**
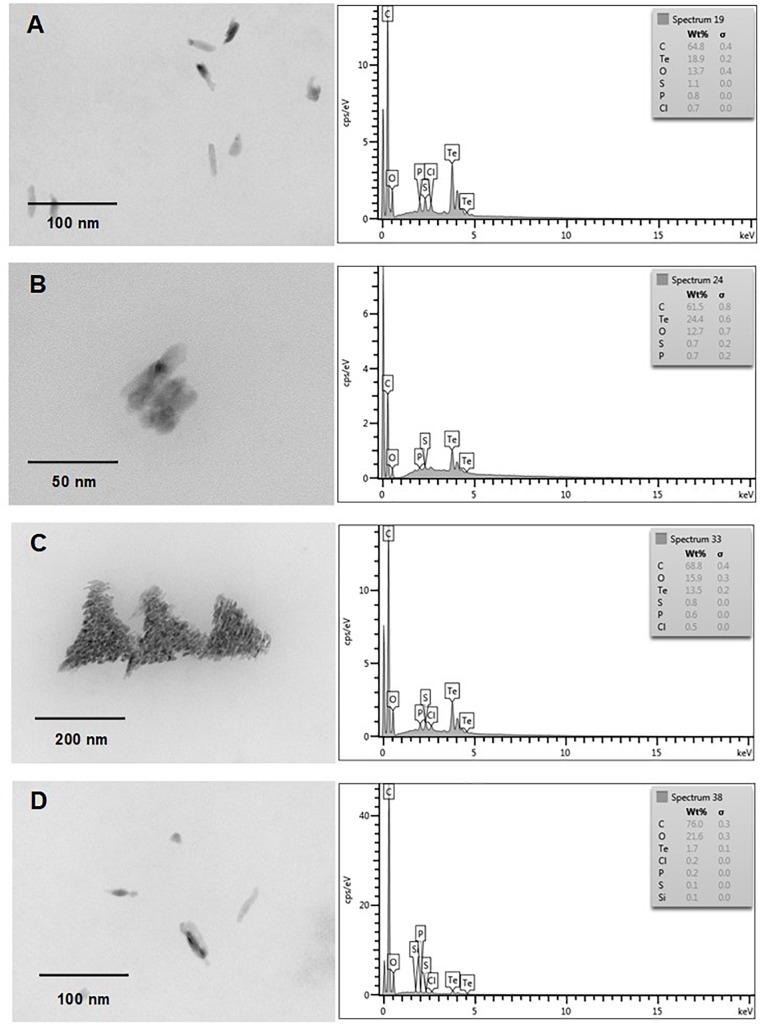
*In vitro*, aerobic synthesis of TeNS by *E. cloacae* MF01 crude extracts. Electron micrographs (left) and EDS analysis (right) show TeNS synthesized under the following experimental conditions: **(A)** NADH**/**pH 8.0; **(B)** NADPH**/**pH 8.0; **(C)** NADH**/**pH 9.0; **(D)** NADPH**/**pH 9.0.

**FIGURE 8:**
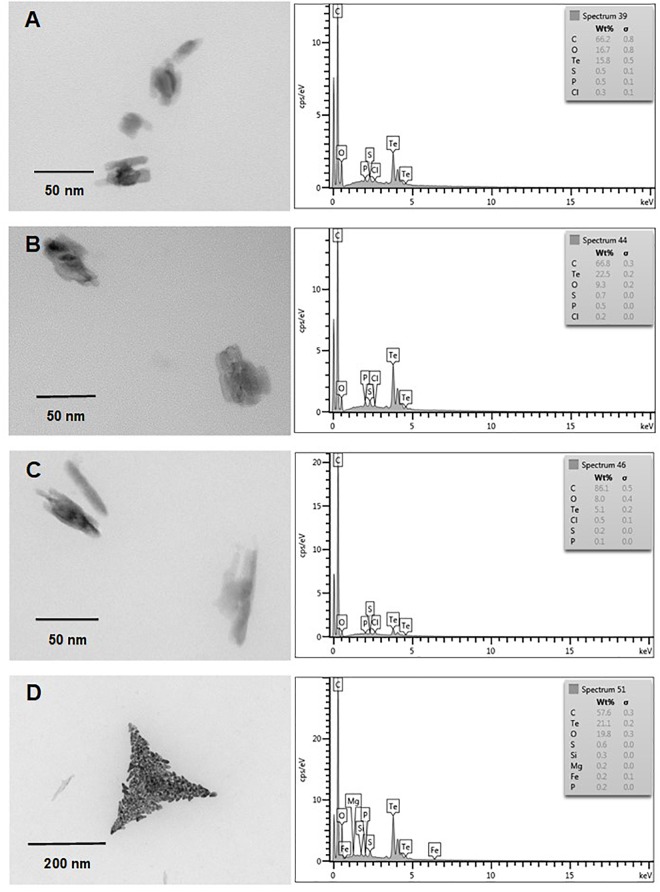
*In vitro*, anaerobic synthesis of TeNS by *E. cloacae* MF01 crude extracts. Electron micrographs (left) and EDS analysis (right) show TeNS synthesized under the following experimental conditions: **(A)** NADH**/**pH 8.0; **(B)** NADPH**/**pH 8.0; **(C)** NADH**/**pH 9.0; **(D)** NADPH**/**pH 9.0.

Finally and independently of the presence of oxygen, TeNS exhibited a uniform, nanostick-like morphology of ∼50 nm. As with AuNS, in some cases aggregates of ∼200 nm were observed (Figures 7C, 8D). Tellurium content of aerobic NS was 18.9, 24.4, 13.5, and 1.7% (Figures 7A–D, respectively). In turn, anaerobically-generated TeNS exhibited a tellurium content of 15.8, 22.5, 5.1, and 21.1% (Figures 8A–D, respectively).

## Discussion

Environmental bacteria thrive in places where growth conditions are very variable, often hostile, such as high or low temperatures, nutrient deprivation, and presence of toxic agents, among others (Bollag et al., 1994). In general, microorganism survival in the presence of toxic agents such as metals and/or metalloids is supported by specific mechanisms that allow them tolerating these agents (Summers and Silver, 1978). Among them, toxicant reduction has caught the attention of researchers, who are using natural or genetically modified metal(loid)-reducing bacteria in the field of bioremediation to decontaminate polluted sites (Stephen and Macnaughton, 1999; Das et al., 2016). In addition, these bacteria are also being employed in biotechnology, since microorganism-mediated metal(loid) reduction often ends in the generation of nanoscaled structures that may exhibit biomedical, electronic, and pharmacological applications (Suresh, 2012).

Although *E. cloacae* MF01 is highly resistant to HAuCl_4_ under aerobic growth conditions, toxicity tests showed that precisely in this condition it contains enhanced ROS levels as well as a decreased RSH concentration. ROS resistance may be related to the antioxidant defense system of this bacterium, i.e., catalase, peroxidase, and superoxide dismutase which eliminate toxic forms of oxygen would protect cell components that are prone to damage such as proteins, cell membranes, and the genetic material (Imlay et al., 1988; Touati et al., 1995; Stadtman and Levine, 2000; Imlay, 2003; Helbig et al., 2008; Hong et al., 2012; Lemire et al., 2013). In this line, while it has been observed that ROS generation is not necessarily related to metal toxicity in *E. coli* (Vrionis et al., 2015), gold exposure results in decreased RSH levels (Shaw, 1999).

*Enterobacter cloacae* MF01 showed higher tellurite resistance in anaerobiosis, where toxicity tests indicated that there was no decrease in RSH concentration or increased ROS generation. It is well-known that one of cell tellurite targets are reduced thiols, which decrease drastically after toxicant exposure (Turner et al., 1999). As RSH are not affected in a ROS-free environment, tellurite toxicity should be lower in anaerobic growth conditions.

After 15 min exposure to HAuCl_4_ or K_2_TeO_3_, most part (90%) of the metal(loid) remains in the extracellular fraction. While tellurite entrance is mediated mainly by the phosphate transporter PitA and/or by the acetate transporter ActP (Borghese et al., 2011; Elías et al., 2012, 2015), that of gold would be mediated by sulfhydryl group-rich membrane proteins (Snyder et al., 1986). As mentioned, about 10% of the toxicants enter the cell, which could generate an adaptive response thus enabling it to better respond to toxicant-mediated damage in aerobic (gold) or anaerobic growth conditions (tellurite).

Intracellular HAuCl_4_- or K_2_TeO_3_-reducing activity from aerobic and anaerobic cultures was analyzed in the pH range 6.0–9.0, using NADH or NADPH as electron donor. Gold and tellurite reductase activities were observed at all pH values assayed irrespective of the electron donor used. Probably pH is responsible for metal speciation, which results in complex formation and/or protonation/deprotonation of amino acids groups that may participate in protein stabilization (Panda and Deepa, 2011). That is the case of proteins exhibiting tellurite reductase activity, whose redox centers contain catalytically important cysteine residues that are sensitive to pH (Arenas et al., 2014). Thiol group deprotonation of vicinal cysteine residues gives rise to the thiolate anion, which is highly reactive exhibiting a pKa ∼8.0 (Vlamis-Gardikas, 2008). Metal(oid)-reducing activity was evaluated only at 37°C, the optimal growth temperature for *E. cloacae* MF01 (Arenas-Salinas et al., 2016), so further optimization of the reducing activity could result from assaying it at different temperatures.

The enzymatic reduction of tellurite generates concomitantly superoxide as a by product, which increases intracellular ROS levels causing oxidative stress and cell damage (Chasteen et al., 2009). In this line, this bacterium exhibits an efficient adaptive response to oxidative stress in aerobic conditions, thus helping enzymes responsible for toxicant reduction to be unaffected (Imlay, 2003). Under anaerobic growth conditions toxicant treatment does not ends in ROS formation, thus reducing protein damage and, therefore, reducing activity (Ercal et al., 2001).

Little is known regarding enzymatic gold reduction. One report states that glutathione reductase from *E. coli* catalyzes HAuCl_4_ reduction in a NADPH-dependent manner (Scott et al., 2008). This contrasts with tellurite reduction, where various bacterial enzymes have been shown to possess the ability to reduce tellurite. These include (i) nitrate reductases (Avazéri et al., 1997), (ii) terminal oxidases of the electron transport chain of several Gram-negative bacteria (Trutko et al., 2000; Díaz-Vásquez et al., 2015), (iii) catalase (Calderón et al., 2006), (iv) isocitrate dehydrogenase and 6-phosphogluconate dehydrogenase from *E. coli* (Reinoso et al., 2012; Sandoval et al., 2015), (v) glutathione reductase from *Pseudomonas* sp. BNF22 (Pugin et al., 2014), (vi) dihydrolipoamide dehydrogenase (Castro et al., 2008; Arenas et al., 2014), thioredoxin reductase and alkyl hydroperoxide reductase from *S. haemolyticus* BNF01, vii) glutathione reductase, flavorubredoxin reductase, dihydrolipoamide dehydrogenase and a putative oxidoreductase from *E. coli* (Arenas-Salinas et al., 2016). In addition, it was recently communicated that glutathione reductase from *E. cloacae* MF01 exhibits tellurite reductase activity (Figueroa et al., 2018).

On the other hand, putative extracellular EPS-mediated HAuCl_4_- and K_2_TeO_3_-reducing activities were also evaluated in the pH range 6.0–9.0. To determine if any protein and/or nucleic acid contributed to the observed activity, EPS preparations were treated with proteases or nucleases prior the reducing assay. A similar mechanism has been described in *E. cloacae* SUKCr1D, where the EPS from this microorganism can reduce Cr(VI) to Cr(III) (Harish et al., 2012).

Extracellular polymeric substance dependent gold-reducing activity was observed only at pH 6.0 and 7.0, in contrast to the results obtained with crude extracts, which revealed gold-reducing activity mainly at pH 8.0 and 9.0. An obvious explanation is that extracellular gold reduction is being carried out by different agents from those forming part of the EPS (Wingender et al., 1999). Since EPS composition is highly variable and depends on the bacterial species and culture conditions, it is very hard to determine the true catalyst(s) (van Hullebusch et al., 2003). In this context, Guibaud et al. (2005) described that purified EPS from *E. cloacae* SUKCr1D is mainly composed of polysaccharides (22 mg/g), proteins (130 mg/g), nucleic- (28 mg/g), and uronic acids (56 mg/g). Our results of EPS-mediated gold-reducing activity support the importance of proteins in the process, since EPS treatment with proteases decreased gold-reducing activity almost to half of that exhibited by untreated EPS irrespective of the presence of oxygen.

Conversely to that observed for HAuCl_4_, no EPS-mediated tellurite reductase activity was found under the tested conditions. Despite this, a white precipitate was always formed. Probably tellurite interacts with positively charged groups that are present in EPS components (i.e., NH_3_^+^ present in amino sugars and proteins), which could chelate the toxicant (Pal and Paul, 2008). A similar result was observed with *E. cloacae* strain P2B EPS, where carboxyl and hydroxyl groups interact with and sequester Pb^2+^ (Naik et al., 2012).

*Enterobacter cloacae* MF01 crude extracts were used to generate metal(loid)-containing NS at the same conditions of pH, enzyme cofactor, and growth conditions that were utilized in metal(loid)-reducing activity tests. In general, reducing activity and NS generation correlated well, excepting for HAuCl_4_-reducing activity from anaerobic cultures at pH 6.0 in the presence of NADH.

AuNS as well as TeNS showed a size < 50 nm, i.e., within the classification of nanostructures (Laurent et al., 2010). While AuNS exhibited circular morphology with irregular borders, TeNS showed a stick-like morphology, commonly referred to as nanosticks. NS size and morphology are parameters of great importance because they control some of the properties the NS exhibit. In fact, the unique physical and chemical properties of NS are dependent on their size, while reactivity, resistance, and other properties are controlled through their size, shape, and structure (Khan et al., 2017). In fact, NS size and morphology is determined during the process of NS formation itself with two crucial stages controlling these parameters: nucleation (the process by which two or more atoms collide to form a cluster) and particle growth (starting when the smallest cluster size reaches the stability at the particular synthesis conditions) (Xiong and Lu, 2015). TeNS synthesized at pH 9.0/NADH/+O_2_ and pH 9.0/NADPH/-O_2_ showed different morphology and smaller size than their counterparts synthesized under the other conditions tested. TeNS appeared as aggregates, which is probably due to size reduction and increased attractive forces that allow NS to interact with each other (Khan et al., 2017). In turn, AuNS exhibited a range of characteristic colors and properties (Dreaden et al., 2012).

Finally and as expected, the chemical composition determined by EDS showed that Au and Te content within NS vary with specific conditions of synthesis. Particularly, one would have anticipated that NS synthesized in anaerobic conditions displayed higher metal(loid) content since metal(loid) oxidation is avoided thus facilitating NS formation (Zare et al., 2013).

## Conclusion

The environmental isolate *E. cloacae* MF01 showed higher resistance to HAuCl_4_ and K_2_TeO_3_ under aerobic and anaerobic growth conditions, respectively. Toxicant’s toxicity was due to ROS generation and RSH depletion. *E. cloacae* MF01 displayed intra- and extra-cellular HAuCl_4_ reduction and only intracellular K_2_TeO_3_-reducing activity. Since in most cases the highest reducing activity did not correlate with higher toxicant resistance, it could be inferred that another resistance mechanism, still unknown, may be involved in alleviating the effects of these toxicants. Finally, the biological synthesis of Au- and Te- containing NS by crude extracts from *E. cloacae* MF01 could represent a start point to support future interesting biotechnological applications.

## Author Contributions

FC, CM-V, CV, and FA conceived and designed the experiments and analyzed the data. FC, EV, KJ, MF, and CM-V performed the experiments. CV and FA contributed reagents, materials, analysis tools, and wrote the paper.

## Conflict of Interest Statement

The authors declare that the research was conducted in the absence of any commercial or financial relationships that could be construed as a potential conflict of interest.
