# Supplementary material for: Reduction of Gold (III) and Tellurium (IV) by Enterobacter cloacae MF01 Results in Nanostructure Formation Both in Aerobic and Anaerobic Conditions
Source: Front Microbiol. 2018 Dec 18;9:3118. doi: 10.3389/fmicb.2018.03118 (PMC6305273; doi:10.3389/fmicb.2018.03118)
Supplement: Supplementary file 1 [file Data_Sheet_1.pdf]

Supplementary Material

Reduction of gold (III) and tellurium (IV) by *Enterobacter cloacae* MF01 results in nanostructure formation both in aerobic and anaerobic conditions

Fernanda Contreras, Esteban Vargas, Karla Jiménez, Claudia Muñoz-Villagrán, Maximiliano Figueroa, Claudio Vásquez and Felipe Arenas

Correspondence to: Felipe A. Arenas E-mails: felipe.arenass@usach.cl

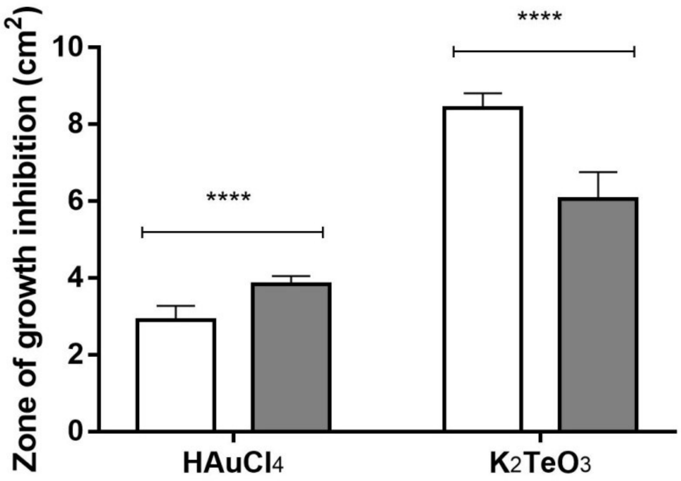

Figure S1. Growth inhibition zones of *E. cloacae* MF01 exposed to $HAuCl_4$ or K_2TeO_3. Zones of growth inhibition in the presence of $HAuCl_4$ (50 mM) or K_2TeO_3 (4 mM) were determined under aerobic (white bars) and anaerobic conditions (grey bars). The data represent the average of 9 independent tests ± SD. ****, $p<0.0001$

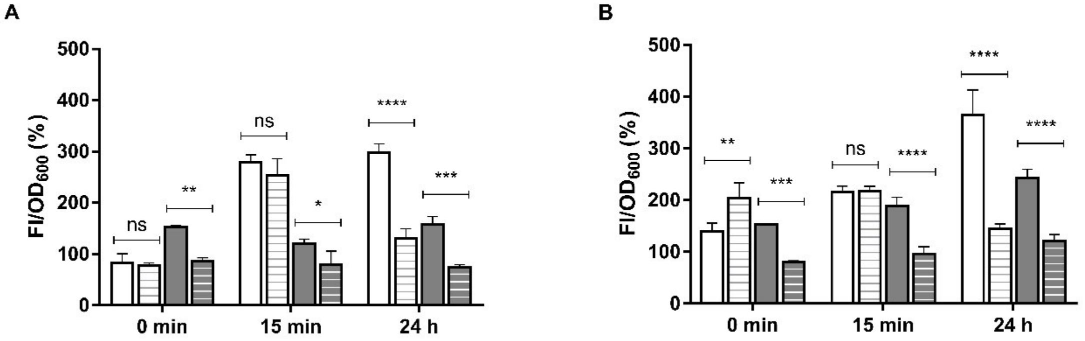

Figure S2. Effect of ascorbic acid on ROS production by *E. cloacae* MF01 exposed to $HAuCl_4$ or K_2TeO_3. Cells were treated for the indicated times with $HAuCl_4$ (**A**) or K_2TeO_3 (**B**) and ascorbic acid, in aerobic (white bars) or anaerobic conditions (gray bars). While open bars represent treatment with $HAuCl_4$ or K_2TeO_3, those filled with lines correspond to toxicant treatment plus ascorbic acid. See legend to Figure 2 for toxicant concentrations. Fluorescence intensity values were expressed as per cent of those observed in the respective control conditions (100%). The data represent the average of 3 independent tests ± SD. *, $p<0.0151$; **, $p<0.0015$; ***, $p<0.0002$; ****, $p<0.0001$; ns, not significant.

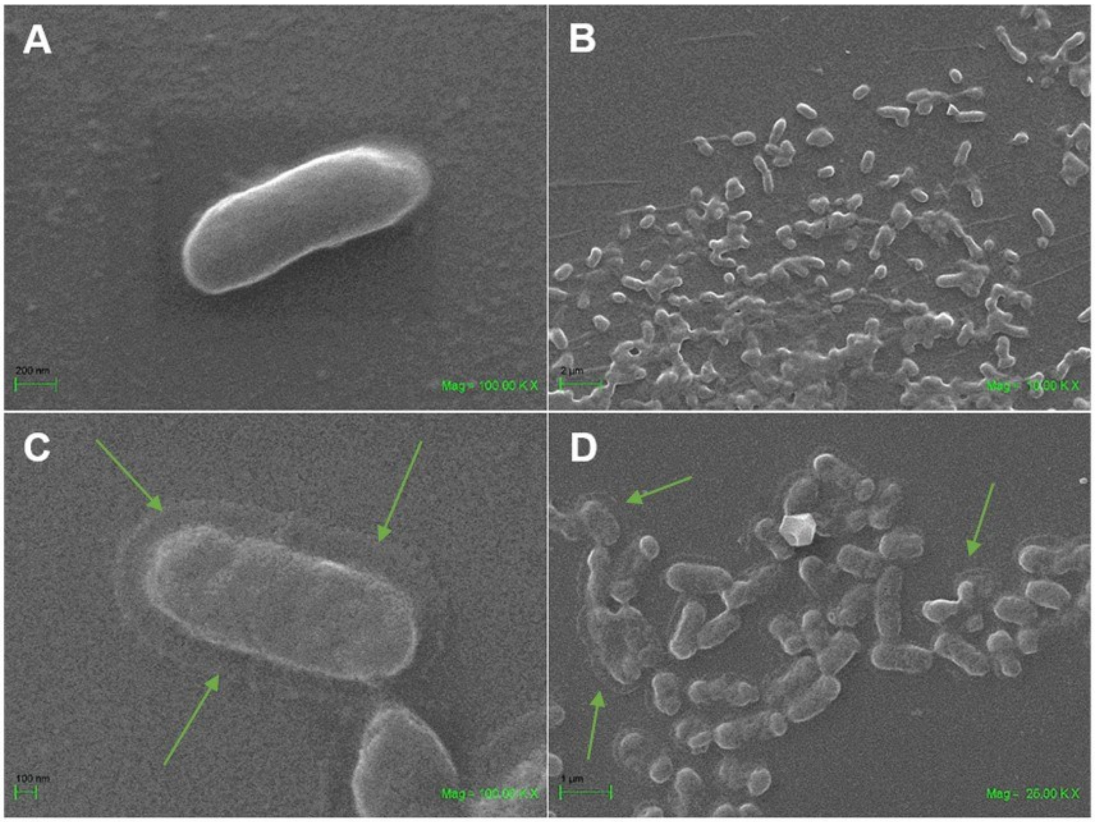

Figure S3. Electron micrograph (SEM) of the environmental isolate *E. cloacae* MF01. Cells were grown in control conditions (no treatment) (**A-B**) and after treating with 80 µM K_2TeO_3 (**C-D**). Green arrows indicate the EPS.

Table S1. Growth parameters of *E. cloacae* MF01 exposed to sub lethal concentrations of $HAuCl_4$ or K_2TeO_3 under aerobic and anaerobic conditions.

	**$HAuCl_4$**			
	Maximal OD_{600}	Lag phase duration (h)	Growth rate (μ)	Generation time (h)
**Aerobic**				
Control	1.66 ± 0.021	0.539 ± 0.061	1.91 ± 0.199	0.366 ± 0.039
7.81 μM	1.55 ± 0.033	3.79 ± 0.318	1.05 ± 0.070	0.661 ± 0.047
15.6 μM	1.51 ± 0.051	9.08 ± 0.675	1.11 ± 0.087	0.902 ± 0.050
31.3 μM	1.33 ± 0.075	14.3 ± 0.416	1.26 ± 0.050	0.553 ± 0.023
**Anaerobic**				
Control	1.24 ± 0.020	0.485 ± 0.034	2.41 ± 0.251	0.290 ± 0.027
7.81 μM	1.17 ± 0.013	0.228 ± 0.072	1.37 ± 0.179	0.512 ± 0.069
15.6 μM	0.986 ± 0.076	2.89 ± 0.444	0.902 ± 0.104	0.778 ± 0.095
31.3 μM	0.917 ± 0.011	5.58 ± 0.594	1.15 ± 0.168	0.614 ± 0.084

	**K_2TeO_3**			
	Maximum OD_{600}	Lag phase duration (h)	Growth rate (μ)	Generation time (h)
**Aerobic**				
Control	1.66 ± 0.008	0.455 ± 0.138	1.97 ± 0.078	0.352 ± 0.014
0.49 μM	1.45 ± 0.026	3.61 ± 0.078	1.51 ± 0.088	0.458 ± 0.027
0.98 μM	1.31 ± 0.101	7.08 ± 0.782	0.967 ± 0.019	1.03 ± 0.020
**Anaerobic**				
Control	1.09 ± 0.026	0.424 ± 0.019	2.18 ± 0.100	0.318 ± 0.015
0.49 μM	1.07 ± 0.006	8.96 ± 0.622	0.749 ± 0.162	0.678 ± 0.164
0.98 μM	0.815 ± 0.150	14.1 ± 1.39	0.868 ± 0.178	0.825 ± 0.159

The data represent the average of 6 independent determinations ± SD.

Table S2. RSH content of *E. cloacae* MF01 exposed to $HAuCl_4$ (0.25 mM aerobic, 0.125 mM anaerobic) and K_2TeO_3 (8 µM aerobic, 0.016 mM anaerobic) under aerobic and anaerobic growth conditions.

	RSH (µM / µg protein)					
	0 min		15 min		24 h	
**Condition**	Control	$HAuCl_4$	Control	$HAuCl_4$	Control	$HAuCl_4$
**Aerobic**	14.51 ± 0.24	8.53 ± 0.06	16.55 ± 0.48	10.05 ± 1.19	21.75 ± 0.11	19.88 ± 2.31
**Anaerobic**	24.56 ± 1.32	26.04 ± 0.98	26.49 ± 0.57	24.50 ± 1.70	13.81 ± 0.42	11.77 ± 0.88

	RSH (µM / µg protein)					
	0 min		15 min		24 h	
**Condition**	Control	K_2TeO_3	Control	K_2TeO_3	Control	K_2TeO_3
**Aerobic**	14.51 ± 0.24	14.25 ± 0.48	16.55 ± 0.48	15.63 ± 0.07	21.75 ± 0.11	22.46 ± 1.50
**Anaerobic**	24.56 ± 1.32	34.47 ± 1.32	26.49 ± 0.57	UD	13.81 ± 0.42	9.75 ± 1.70

The data represent the average of 3 independent determinations ± SD.

Table S3. Concentration of AuNS and TeNS synthesized under optimal reduction conditions

	AuNS (μg/mL)		TeNS (μg/mL)	
NADH	$+O_2$	$-O_2$	$+O_2$	$-O_2$
pH 8.0	-	1600	400	200
pH 9.0	900	400	400	400
NADPH	$+O_2$	$-O_2$	$+O_2$	$-O_2$
pH 8.0	400	-	500	100
pH 9.0	300	200	700	200
